# Supplementary material for: Formation of a swelling gel underlies a morphological transition in Bacillus subtilis biofilms
Source: bioRxiv. 2026 Feb 22:2026.02.20.707077. Preprint. [Version 2] doi: 10.64898/2026.02.20.707077 (PMC12934947; doi:10.64898/2026.02.20.707077)
Supplement: Supplement 7 [file NIHPP2026.02.20.707077v2-supplement-7.pdf]

bioRxiv preprint doi: https://doi.org/10.64898/2026.02.20.707077; this version posted February 22, 2026. The copyright holder for this preprint (which was not certified by peer review) is the author/funder, who has granted bioRxiv a license to display the preprint in perpetuity. It is made available under aCC-BY 4.0 International license.

Supplementary Information

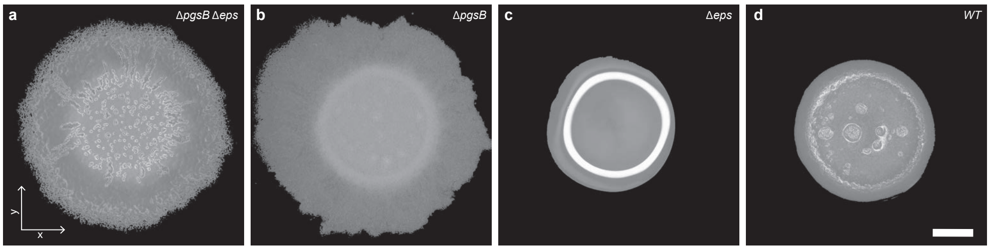

Fig. S1 Top-down stereoscopic images of 24-hour-old biofilms formed by strains with differential production of PGA and EPS polymers a, Δ*pgsB*Δ*eps* (neither polymer), **b,** Δ*pgsB* (EPS only), **c,** Δ*eps* (PGA only), **d,** WT (both polymers). The bright ring in **c** is a reflection of the overhead illumination light due to the excessively shiny surface of Δ*eps* colonies.

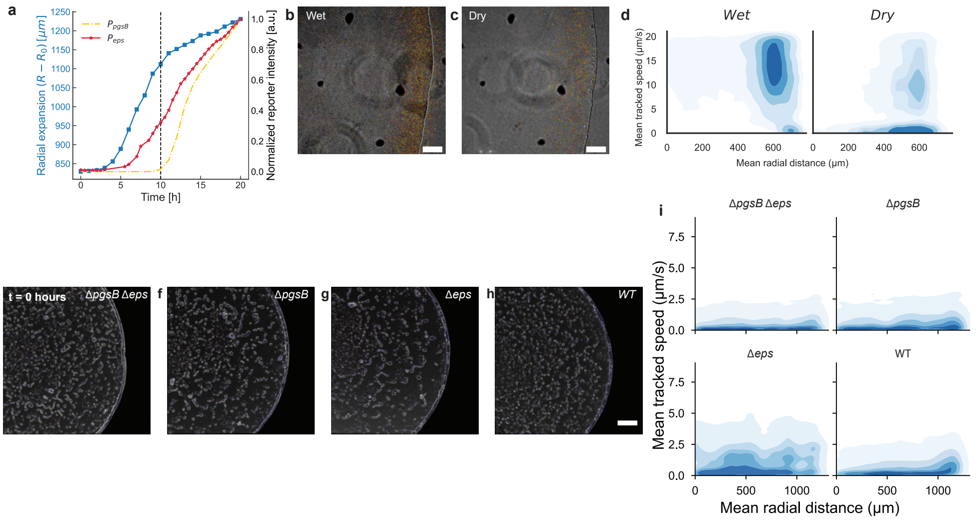

Fig. S2 Reporter dynamics and spatial distribution of cell speeds in biofilms (a, e–i) and in a liquid droplet (b–d) during the wet–dry transition a, Colony radius (blue) of the wild-type (WT) strain over 20 hours of growth, together with constitutive reporter activity (P_{pen}, red) and the reporter for the PGA biosynthesis gene *pgsB* (P_{pgsB}, yellow). Signal from the *pgsB* reporter emerges at $t \approx 10$ hours. **b,c,** Phase-contrast images (20×) of WT droplets immediately after spotting in the wet state (**b**) and following drying (**c**). Scale bar, 100 μm. **d,** Kernel density estimates (KDEs) of mean tracked cell speeds and radial trajectories in wet versus dry droplets reveals a shift from nonzero velocities in the wet state to near-zero speeds upon drying. **e-h,** Phase-contrast images (10×) of Δ*pgsB* Δ*eps*, Δ*pgsB*, Δ*eps* and WT at $t \approx 0$ hour. Scale bar, 200 μm. **i,** Kernel density estimates (KDEs) of mean tracked cell speeds and radial trajectory distributions at $t \approx 0$ hour show uniformly scattered, low-speed motion across all strains.

bioRxiv preprint doi: https://doi.org/10.64898/2026.02.20.707077; this version posted February 22, 2026. The copyright holder for this preprint (which was not certified by peer review) is the author/funder, who has granted bioRxiv a license to display the preprint in perpetuity. It is made available under aCC-BY 4.0 International license.

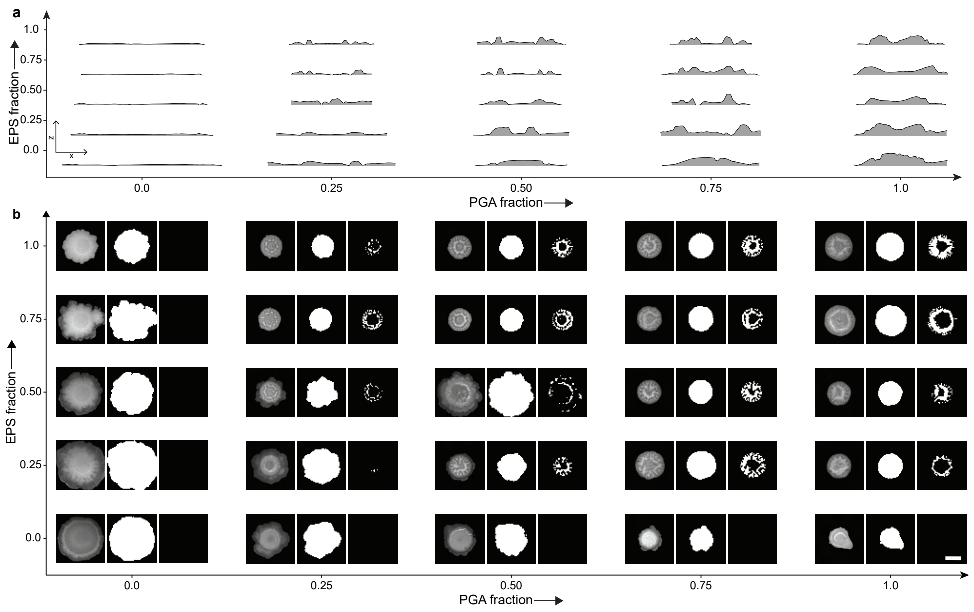

Fig. S3 Phase diagrams of segmented OCT xz profiles and wrinkle/biomass masks a, Phase diagram of segmented surfaces from optical coherence tomography (OCT) xz-profiles of biofilms with increasing fractions of PGA and EPS–producing cells (0–100%). Scale bar 1 mm. **b**, Phase diagram showing wrinkling coefficient analysis workflow - stereoscopic grayscale image and the corresponding biomass and wrinkle masks(left to right).

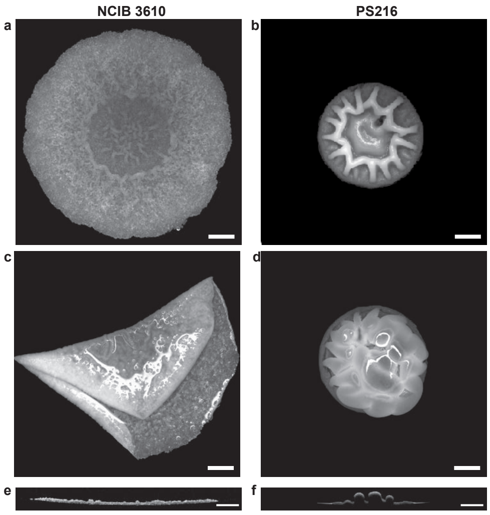

Fig. S4 Top-down stereoscopic images and OCT xz profiles of NCIB3610 and PS216 biofilms before and after water immersion a-d, Top-down stereoscopic images of 48-hour-old *Bacillus subtilis* biofilms formed by strains NCIB3610 and PS-216 before (**a, b**) and after water immersion (**c,d**). Scale bar, 2 mm. NCIB3610 does not produce PGA under standard biofilm conditions due to a mutation in the plasmid-borne gene *rapP*. PS216 naturally lacks the pBS32 plasmid that carries *rapP*. We expect it to behave similarly to our WT, 3610 pBS32^0. **e, f** Corresponding xz profiles measured by optical coherence tomography. Scale bar, 1 mm.

bioRxiv preprint doi: https://doi.org/10.64898/2026.02.20.707077; this version posted February 22, 2026. The copyright holder for this preprint (which was not certified by peer review) is the author/funder, who has granted bioRxiv a license to display the preprint in perpetuity. It is made available under aCC-BY 4.0 International license.

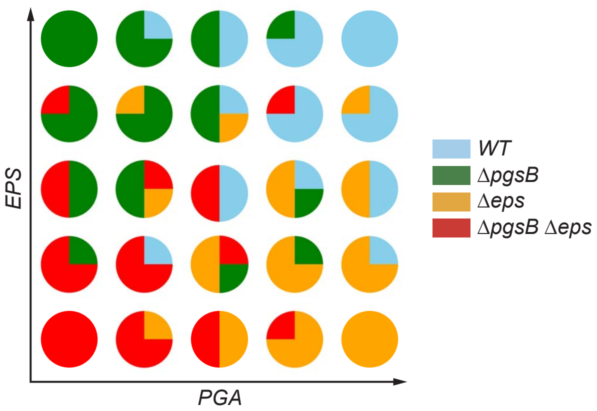

Fig. S5 Pairwise combinations of four Bacillus subtilis strains visualized as pie charts representing their relative contributions in co-culture and co-co-culture assays. Each pie chart depicts the proportion of two/three strains in the mixture, with colors corresponding to strain identities: WT (light blue), Δ*pgsB* (green), Δ*eps* (gold), and Δ*pgsB* Δ*eps*(red). The x-axis (PGA) and y-axis (EPS) indicate increasing producer fractions of produce poly-γ-glutamate or exopolysaccharide, respectively.

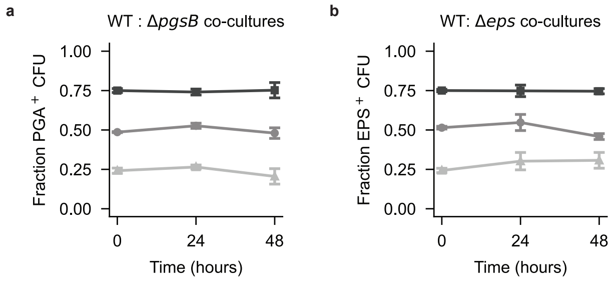

Fig. S6 Proportion of PGA-producing and EPS-producing CFUs over time in co-culture biofilms . a, b Temporal evolution of PGA$^+$ CFU fraction in a co-culture of WT : Δ*pgsB* (**a**) and of EPS$^+$ CFU fraction in a co-culture of WT : Δ*eps* (**b**) with three different initial genotypic ratios of Δ*pgsB* and Δ*pgsB* Δ*eps* cells, 0.75:0.25 (squares), 0.5:0.5 (circles), and 0.25:0.75 (triangles). Points represent average ± S.D. from three independent replicates ($n = 3$).

Supplementary Videos

- Video V1: Δ*pgsB* Δ*eps* 10X phase time-lapse video at t = 10 hours.
- Video V2: Δ*pgsB* 10X phase time-lapse video at t = 10 hours.
- Video V3: Δ*eps* 10X phase time-lapse video at t = 10 hours.
- Video V4: WT 10X phase time-lapse video at t = 10 hours.
- Video V5: Wet droplet 20X phase time-lapse video.
- Video V6: Dry droplet 20X phase time-lapse video.

bioRxiv preprint doi: https://doi.org/10.64898/2026.02.20.707077; this version posted February 22, 2026. The copyright holder for this preprint (which was not certified by peer review) is the author/funder, who has granted bioRxiv a license to display the preprint in perpetuity. It is made available under aCC-BY 4.0 International license.

Thin-film bilayer model

We modeled biofilms as thin films of Young's modulus E_f adhered to agarose substrates of Young's modulus E_s [7, 36]. For such a bilayer system under strain, film wrinkling will occur beyond a critical strain measured relative to the stress-free configuration $\epsilon_c \sim (E_s/E_f)^{2/3}$ [37–39, 52]. Because all the biofilms were grown on 2% agarose substrates, we assume that E_s is a constant. Thus, the critical strain necessary for the emergence of wrinkles in this simplified model is solely modulated by the biofilm stiffness E_f. For a biofilm to wrinkle, the strain, ϵ, must exceed ϵ_c. PGA or EPS could cause wrinkling by either increasing ϵ or decreasing ϵ_c (via E_f). In our experiments, PGA causes water absorption and swelling without a significant impact on the extent of dissolution in water (Fig. 3, Fig. 4), while EPS prevents dissolution without causing significant swelling (Fig. 2, Fig. 4). For these reasons, we assume that strain depends primarily on the fraction of PGA-producers, f_{PGA}, and that stiffness depends primarily on the fraction of EPS-producers, f_{EPS}. In the following sections, we discuss how PGA-induced swelling modulates in-plane strain and EPS-driven gelation modulates the critical strain necessary for the emergence of wrinkles.

PGA-induced isotropic swelling gives rise to in-plane strain

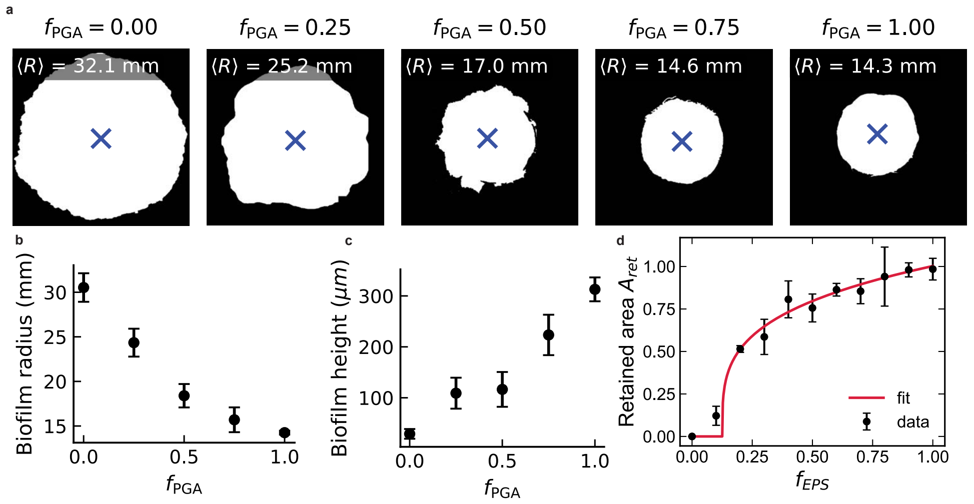

Fig. S7 Representative binary masks, colony metrics versus PGA fraction and retained area versus EPS fraction a, Representative binary masks of biofilm colonies at increasing PGA fractions (f_{PGA}), showing colony centroids (blue crosses) and mean colony radii $\langle R \rangle$. **b,** The mean biofilm radius R decreases as f_{PGA} increases. **c,** The maximum biofilm height h increases monotonically with f_{PGA}. **d,** Retained area A_{ret} as a function of EPS fraction f_{EPS}, showing experimental data (black circles, mean ± S.D. from three independent replicates ($n = 3$)) and fit to the theoretical model $(f_{EPS} - f_c)^{\beta}$ with $\beta = 0.271 \pm 0.114$ and $f_c = 0.126 \pm 0.078$ (red line).

Through our experiments, we demonstrate that PGA production promotes fluid uptake from the underlying substrate, resulting in a monotonic increase in the biofilm's maximum height with the fraction of PGA-producing cells. Concurrently, we observe a reduction in the base radius as the concentration of PGA polymers increases (Fig. S7a-c). To interpret how these morphological changes generate in-plane strain, we propose a simple physical model: envision water being injected into a sponge whose base is constricted and moderately adhered to the substrate, causing isotropic swelling. In this scenario, an increase in water volume, together with the fixed base, produces an in-plane compressive strain. Assuming that the volume of absorbed fluid – and hence

bioRxiv preprint doi: https://doi.org/10.64898/2026.02.20.707077; this version posted February 22, 2026. The copyright holder for this preprint (which was not certified by peer review) is the author/funder, who has granted bioRxiv a license to display the preprint in perpetuity. It is made available under aCC-BY 4.0 International license.

the strain – is a monotonically increasing function of the fraction of PGA polymers (f_{PGA}), we can express the strain with the following scaling relation:

$$\epsilon \sim f_{PGA}^{\alpha}. \tag{2}$$

We require the power law exponent $\alpha > 0$ so that ϵ increases with increasing f_{PGA}, but leave its value unspecified to maintain generality. The key assumption made here is that the EPS polymer does not contribute to the in-plane strain. Based on our data, this appears to be a reasonable assumption because neither the height nor the biofilm footprint, indirect indicators of in-plane strain, are found to change significantly when the EPS fraction is varied in experiments at a fixed PGA fraction.

EPS drives sol-gel phase transition via cross-linking

In our system, EPS-producing cells generate cross-linking EPS polymer that drives a gelation phase transition from a sol state at low EPS fractions to a gel state at high concentrations of EPS polymers. As shown in Fig. 4e, addition of water to the biofilm reveals a phase transition in which the fraction of retained biofilm area, a proxy for gel fraction P_{gel} i.e., fraction of connected EPS clusters, increases sharply and follows a power law:

$$A_{ret}(f_{EPS}) \sim (f_{EPS} - f_c)^{\beta}, \;\; f_{EPS} > f_c \tag{3}$$

with experimentally determined values $\beta = 0.271 \pm 0.114$ and $f_c = 0.126 \pm 0.078$ by fitting the area retained on water addition as a function of f_{EPS} plot to the proposed power law (Fig. S7d). Below the threshold, the biofilm fails to resist dissolution, indicating the absence of a coherent gel network.

Gelation is often modeled as a percolation transition [32]. A key result from these theories is that the emergence of mechanical rigidity in polymer networks is intimately linked to the formation of a system-spanning cluster of cross-links. Theoretical and experimental studies of polymer gels and percolation networks have established that the onset of elasticity coincides with the formation of the percolating cluster that marks the gel point [53, 54]. In these models, the elastic modulus emerges only above the critical fraction f_c where long polymer chains get strongly cross-linked, forming an elastic gel material. Below the threshold f_c, the material behaves like a liquid, having little to no elasticity. Hence, we expect the Young's modulus E_f to follow a scaling relation:

$$E_f \sim (f_{EPS} - f_c)^{\mu}, \;\; f_{EPS} > f_c, \tag{4}$$

where the exponent μ characterizes the steepness of the modulus growth near the threshold and is typically expected to be larger than β [55, 56]. The power-law scaling of Young's modulus above the EPS fraction threshold f_c is a well-established feature of percolation and gelation transitions, and is consistent with our measurements of retained biofilm area. In three dimensions, the elasticity scaling exponent μ takes values of 3 in the mean-field model, 1.8 in the electrical analogy model [57], and 3.7 in the bond bending model [58]. Experimentally, μ has been observed to extensively vary, with values near 1.7 for gelatin [59], ~ 1.9 measured in diisocianate-triol gels [60] and approaching 3 in polyesters [61]. Accordingly, we consider a wide range of critical exponents $\mu \approx 1.5$-4, to test the robustness of our model. The key assumption made here is that the stiffness modulus E_f of the biofilm scales independently of the PGA fraction f_{PGA}. This is consistent with our observation that the Δeps strain, which is devoid of any EPS polymer and solely has PGA polymer producers(PGA$^+$, EPS$^-$), dissolves in water and behaves like a sol because it lies below the critical crosslinking threshold f_c necessary for the sol-gel transition.

bioRxiv preprint doi: https://doi.org/10.64898/2026.02.20.707077; this version posted February 22, 2026. The copyright holder for this preprint (which was not certified by peer review) is the author/funder, who has granted bioRxiv a license to display the preprint in perpetuity. It is made available under aCC-BY 4.0 International license.

Phase boundary between wrinkled and smooth surface morphology

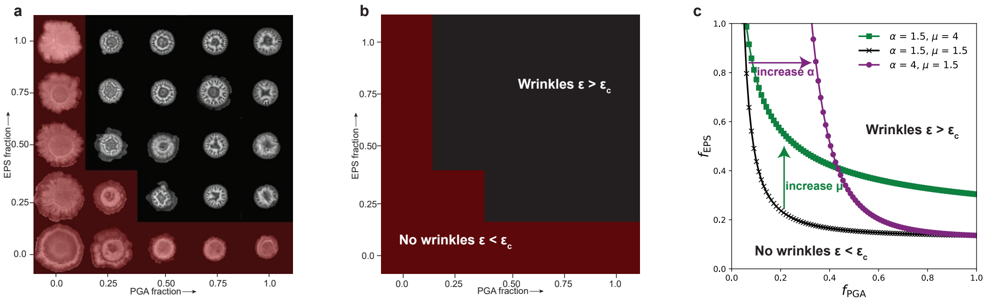

Fig. S8 Comparision between experimental and model phase portraits a, Phase portrait of biofilm morphologies across PGA and EPS fractions, showing representative images for each condition, overlaid with a red layer for the smooth biofilms. **b,** Binary phase portrait generated from the model with $\alpha = 2$, $\mu = 1.5$, $f_c = 0.126$ and $K = 0.01$. **c,** Phase boundaries obtained from the model for a range of parameters α and $\mu \in [1.5, 4]$

.

Combining these relationships between the PGA swelling-induced strain (eq. 2), the EPS-driven gelation phase transition and stiffening (eq. 4) and exploiting the equation for critical strain necessary for wrinkling in the elastic bilayer model ($\epsilon_c \sim E_f^{-2/3}$), we can predict the boundary where biofilms transform from smooth to wrinkled, i.e., where the strain ϵ generated by swelling from the PGA-producers at fraction f_{PGA} equals the critical strain ϵ_c, which in turn is determined by the fraction f_{EPS} of EPS polymers. At this boundary, we rewrite the expression for strain in terms of the f_{PGA} and that for Young's modulus in terms of f_{EPS} and the other model parameters f_c, α , and μ to obtain the equation for the phase boundary as follows:

$$f_{PGA}^{\alpha} \sim (f_{EPS} - f_c)^{-2\mu/3} \tag{5}$$

$$\implies f_{EPS} = K f_{PGA}^{\frac{-3\alpha}{2\mu}} + f_c. \tag{6}$$

Here K is a proportionality constant. Fig. S8a, b shows an agreement between the experimental wrinkling phase portrait and the one derived from the model by setting $\alpha = 2$, $\mu = 1.5$ and $K = 0.01$. Fig. S8c shows a qualitative agreement of our experimental result with this theoretical model over a wide range of model parameters α and μ.
